# Using Acoustics to Determine Eelgrass Bed Distribution and to Assess the Seasonal Variation of Ecosystem Service

**DOI:** 10.1371/journal.pone.0150890

**Published:** 2016-03-08

**Authors:** Shiori Sonoki, Huamei Shao, Yuka Morita, Kenji Minami, Jun Shoji, Masakazu Hori, Kazushi Miyashita

**Affiliations:** 1 Graduate School of Environmental Science, Hokkaido University, 20-5 Benten-cho Hakodate-city, Hokkaido, Japan; 2 Institute for East China Sea Research, Graduate School of Fisheries Science and Environmental Studies, Nagasaki University, 1551-7 Taira-cho Nagasaki-city, Nagasaki, Japan; 3 Graduate School of Fisheries Science, Hokkaido University, 3-3-1 Minato-cho Hakodate-city, Hokkaido, Japan; 4 Center for Field Science of the Seto Inland Sea, Hiroshima University, 5-8-1 Minato-cho Takehara-city, Hiroshima, Japan; 5 National Research Institute of Fisheries and Environment of Inland Sea, Fisheries Research Agency, Maruishi 2-17-5, Hatsukaichi-city, Hiroshima, Japan; 6 Field Science Center for Northern Biosphere, Hokkaido University, 20-5 Benten-cho, Hakodate-city, Hokkaido, Japan; Università della Calabria, ITALY

## Abstract

Eelgrass beds are an important source of primary production in coastal ecosystems. Understanding seasonal variation in the abundance and distribution of eelgrass is important for conservation, and the objectives of this study were to 1) monitor seasonal variation in eelgrass beds using an acoustic monitoring method (Quantitative echo sounder) and 2) broadly quantify the carbon circulation function. We obtained acoustic data of eelgrass beds in coastal areas north and east of Ikunojima Island. Surveys were conducted nine times over the 3-year period from 2011 to 2013 in order to monitor seasonal variation. Acoustic data were obtained and used to estimate the spatial distribution of eelgrass by geostatistical methods. To determine supporting services, we determined carbon sink and carbon fixation by eelgrass beds using data from the National Research Institute of Fisheries and Environment of Inland Sea (2011). The height and distribution of eelgrass beds were at a maximum in May and at a minimum in November of each year. Distribution trends were different between the north and east areas. Supporting services showed the same patterns throughout the year. The area of distribution was considered to be coincident with the life history of eelgrass. Distribution differed by area and changed yearly due to the effects of bottom characteristics and wind direction. Quantifying the supporting services of eelgrass beds was shown to be useful for managing the conservation of coastal ecosystems.

## Introduction

Eelgrass (*Zostera marina* L.) is found in inner bays with a water depth of less than 10 meters and sand or clay substrate. Eelgrass beds grow thickly, reaching high densities [1]. Eelgrass grows most quickly in spring and declines from summer to autumn, and increases again after winter in coastal waters of Japan from Hokkaido to Kyushu. Eelgrass beds are known to be important as areas of high primary production in coastal ecosystems. Eelgrass beds provide various ecosystem services, such as primary production through carbon fixation from photosynthesis [2], nutrient cycling in the region above the sea bottom [3], as a spawning ground of fishes [4], habitation and hiding place of juvenile fishes and larvae and [5] recreation areas including for fishing. Coastal area including eelgrass beds is the effective sea area for human. According to Millennium Ecosystem Assessment [6], the human well-being of coastal inhabitants is on average much higher than that of inland communities. Coastal areas are assumed to provide about 30% of ecosystem services on Earth, although their total area is small [7]. In particular, the ecosystem services of eelgrass beds are as much as three times greater than that of coral reefs. However, research on the ecosystem service of eelgrass beds has been far less than that for coral reefs and mudflats, and knowledge about eelgrass beds has been limited until now [8]. In the coastal waters around Japan, eelgrass beds declined about 40% over 30 years, due to development and changes in the environment [9]. Ecosystem services of eelgrass beds are varied in function and show seasonal variation [10]. The ecosystem services of eelgrass beds and the supporting services are typified by nutrient cycling functions, such as carbon sink and carbon fixation. Through production, organic substances are removed from the sea water and assimilated by photosynthesis, producing a carbon sink. Carbon fixation is the amount of organic carbon that is transferred from the eelgrass to the sea bottom, as through decomposition on the sea bottom. Eelgrasses that sink to the bottom are decomposed and are fixed in the in sea as organic carbon. This organic carbon supports a larger number of microorganisms and higher trophic levels. Thus, eelgrass supports greater biodiversity of the sea ecosystem. Understanding the amount of carbon sink and fixation in eelgrass beds are necessary to keeping biodiversity in the sea area. The ecosystem services of eelgrass beds are primarily calculated as amounts of mass assimilated by unit area or gram dry weight (gdw) [11, 12], but there are few comprehensive studies have been conducted. Further information on the distribution of eelgrass in each season is needed in order to determine basic seasonal variation and biomass of eelgrass beds, which will help to understand the importance of eelgrass beds [13, 14].

In previous studies, direct and remote sensing methods were used to estimate the distribution of eelgrass beds. Direct sensing is typically conducted using the quadrat method [15] and diving [16]. These methods have the advantage being able to make direct observations of eelgrass and of directly determining the distribution. However, conducting studies by these methods demands too much physical energy and time for monitoring, and the accuracy of observations tends to deteriorate for observations made at deeper monitoring points. Remote sensing is typified by aerial and satellite photography [17]. These methods can be used to monitor the distribution of eelgrass beds on a wide geographic scale, but obtaining this data is expensive and the range of visualization can be limited due to turbidity, depth and weather conditions. Thus, we seek to establish methods for collecting data that are efficient, sustainable, and repeatable and that can be applied over a wide geographical range in order to assess the wide range of seasonal variation of eelgrass beds.

Ultrasound pulses can be used to visualize underwater, such as the sea bottom, schools of fish or sea water. Acoustic methods using quantitative echo sounders can be used to understand the standing stock and biology of various fish and plankton. Recently, this method was applied to surveys of eelgrass beds [18]. Ultrasound pulses reflect differently from substances, such as sea water; the strongest reflection is off of the swim bladder because it contains air [19]. As eelgrass has lacunae in the inner leaves [1], we can recognize eelgrass beds based on the strong reflections of ultrasonic pulses [20]. Acoustic data can visualize the continuous sea bottoms and undersea structures under the boat, because data were continuously collected with survey boat moving. It becomes easy to understand whether there are eelgrass beds or not from acoustic and GPS data.

The objective of this study was to make a rapid and quantitative estimation of the distribution of eelgrass beds, including seasonal variation. The methods include acoustic sensoring, and quantification of carbon sinks and fixation, which are supporting services. The study was conducted in the coastal areas near Ikunojima Island off Takehara City, Hiroshima Prefecture in the Seto Inland Sea. This area was selected because the eelgrass beds in this area are in a natural state and few developments, such as bank protection work have been undertaken. The environment of this coastal area is varied, partly due to the large tidal range in the Seto Inland Sea. The North area is an inner bay with a flat sea bottom and shallow depth of about 3 to 4 m, and the East area is an open coastal area with a steep pitch of the sea bottom. Eelgrasses are found in both areas; it grows prolifically in the North area but less so in the East area. In order to easily understand the ecosystem service of eelgrass beds under natural conditions, we calculated the carbon sink and fixation, which is part of the supporting services of eelgrass beds, based on distribution determined using acoustic method.

## Materials and Methods

**Fig 1:**
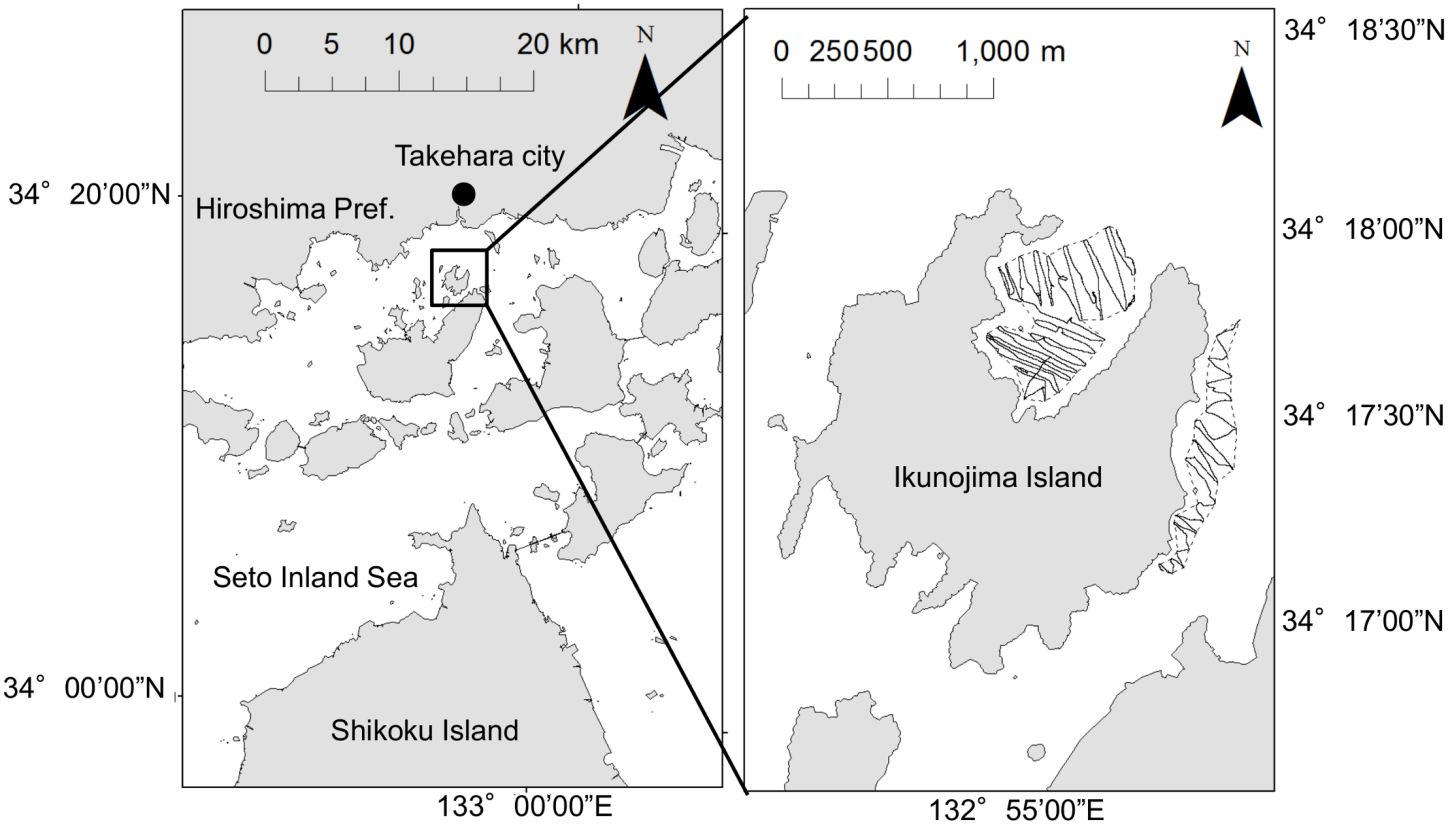
Study areas in this study are located adjacent to Ikunojima Island, off Takehara City, Hiroshima Prefecture. In the inset area, the survey areas are outlined with dashed lines and the survey lines are indicated with solid lines. Map of survey area was obtained from Geospatial Information Authority of Japan (http://www.gsi.go.jp/) and processed [21].

The study areas are located in the coastal waters near Ikunojima Island (Fig 1): North area (0.33 km^2^) and East area (0.15 km^2^).

We conducted this survey in the boat by permission of the Hiroshima University. This survey area is not protected region. The field studies did not involve endangered or protected species. Transect lines were set at intervals of 10 to 30 m in the survey areas. Surveys were conducted by boat nine times: November 2011 and May, July, September and November in 2012 and 2013. On each transect, the boat was stopped at random points to check the growth condition of eelgrass using an underwater camera and measured water temperature and salinity using a compact CTD (JFE Advantech Co., Japan). All surveys were conduct in daytime.

### Acoustic data

Acoustic data were collected along each transect using a small quantitative echo sounder KCE-300 with split beam transducer (120 kHz, Sonic Co., Japan, Table 1) at depths down to 20 m. Eelgrass release gas into water which was produced by photosynthesis. There is a possibility that eelgrass can’t be correct evaluation as the resonance of small gas in the seawater when using low frequency transducer [22]. Thus, we need to use high frequency transducer as possible to prevent resonance. In this study, the transducer with 120kHz frequency was used to conduct survey as which is small, easy to be attached and released from outside board of boat. Eelgrass beds presence was extracted from the acoustic data using Echoview4.9 (Myriax, Australia).

**Table 1:** Specification of quantitative echo sounder, KCE-300 with T182 transducer (Sonic Co.).

KCE-300 with a T182 transducer	
Frequency (kHz)	120
Pluse length (ms)	0.6
Beam width (degree)	8.5
Resolution (cm)	3.5
Ping rate (s^-1^)	5
Beam type	Split beam
Weight of transducer (kg)	8.0
Diameter of transtuder (cm)	13.0

**Fig 2:**
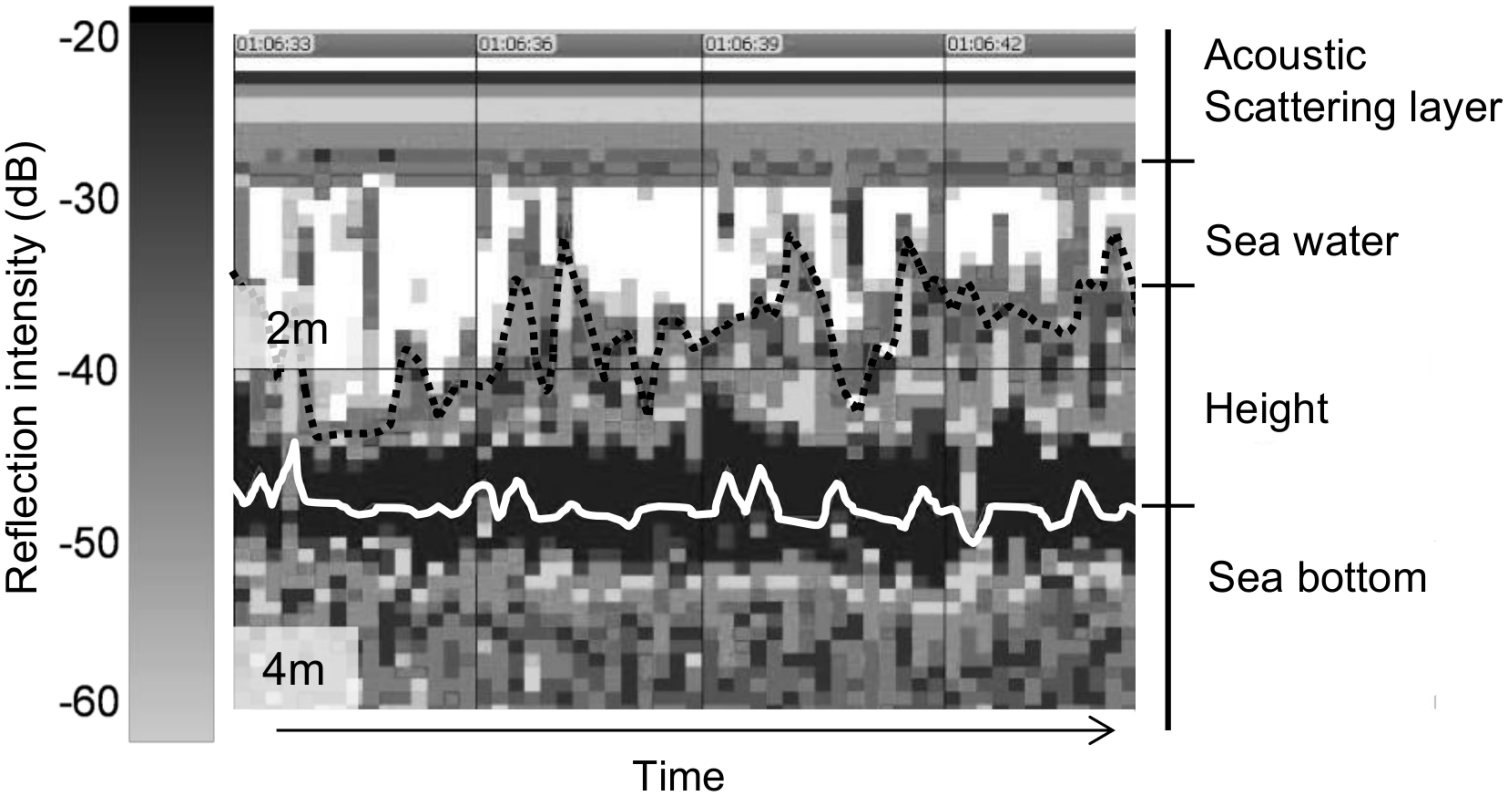
Representative echogram of an eelgrass bed. The color bar at the left indicates the reflection intensity. Black solid line represents the acoustic scattering layer, black dashed line represents the upper edge of eelgrass beds, white solid line indicates the sea bottom and lower sides of eelgrass beds. X-axis indicates time and Y-axis indicates depth. Travelling direction of boat is right side.

Reflection intensity was set to recognize three types of surfaces, sea water, eelgrass beds and sea bottom (Fig 2).

**Fig 3:**
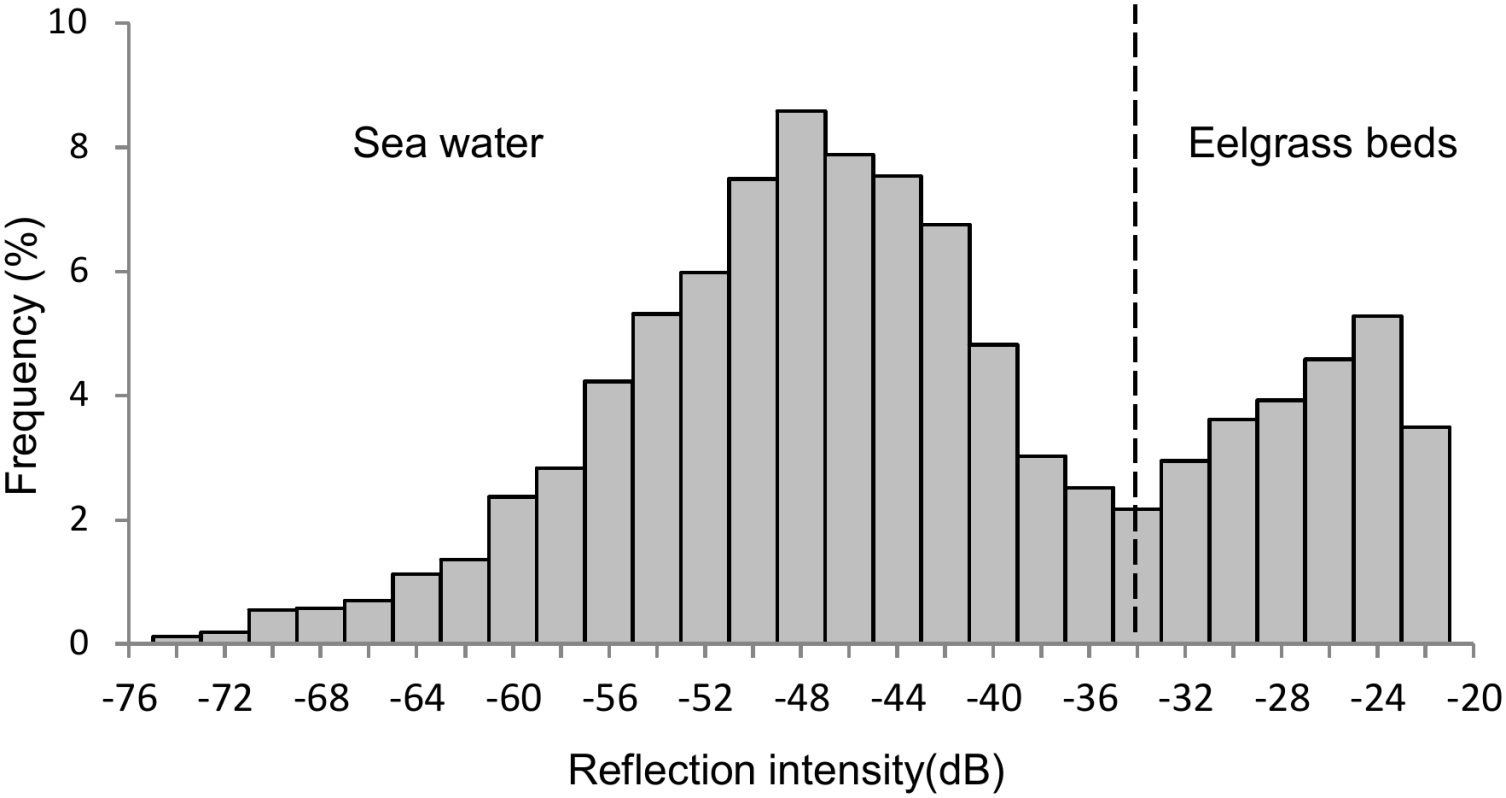
Histogram of reflection intensity showing the set threshold between sea water and eelgrass beds. The threshold was determined for each set of survey, and this figure shows the threshold set in November 2013. All acoustic threshold shows in S1 Dataset.

We excluded the boundary of the acoustic scattering layer [23] from analysis. In this study, the dead zone near sea bottom, which is impossible detect because of the pulse length [24], was at 45 cm and the data collected under this depth was also excluded. The threshold between the sea bottom and the eelgrass beds was defined as having the strongest reflection. To extract the presence of eelgrass beds from the echogram, we made a histogram of the reflective intensity and defined the backscattering strength of the eelgrass beds. First, we extracted the reflective intensity at five random positions where the eelgrass was present. The horizontal range was set to a vertical 20 m column, excluding the acoustic scattering layer and the dead zone. Then, we made a histogram of reflective intensity every 2 dB at each position. The histogram showed bimodal reflection intensity: sea water and eelgrass beds. The average value of the lowest frequency was taken as the boundary between the two modes and the upper threshold of the eelgrass beds. This threshold was set based on histogram of reflection intensity data in each survey (Fig 3), because reflection intensity varied as leaves density in eelgrass beds is different of each season [25]. The average threshold of reflection intensity between the eelgrass beds and sea water was -46.4dB.

**Fig 4:**
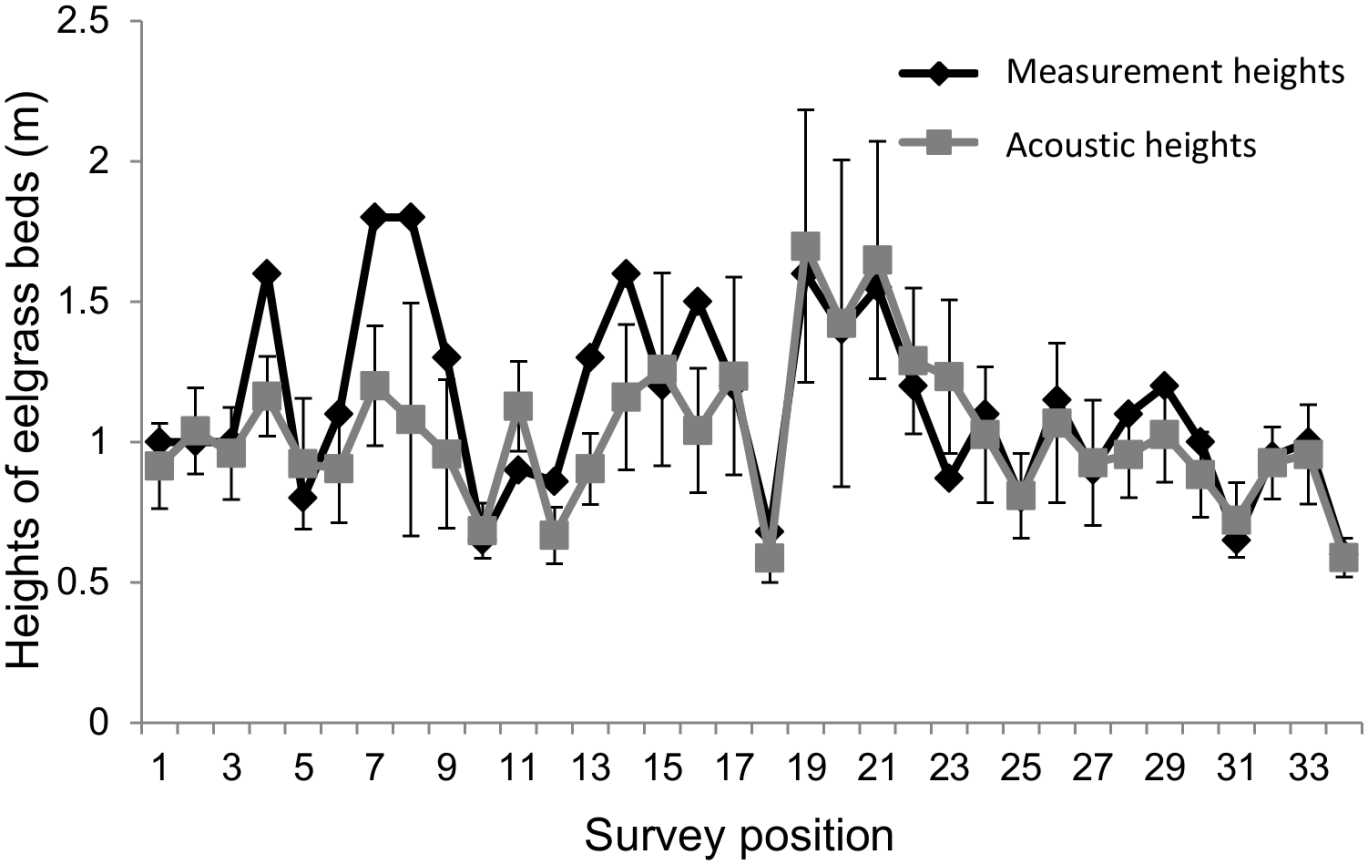
Heights of eelgrass beds by direct measurement and acoustic method in each measurement position. These data were collected during July, 2012 and Sep. 2013, measurement position was randomly selected (S2 Dataset). Dark gray is value by direct measurement, and light gray is acoustic value. Error bars indicate standard deviation of acoustic value.

At 2m intervals, we extracted location information from acoustic data, and calculated the height of eelgrass beds, which is defined as the distance between the sea bottom and the top of eelgrass beds in a flourishing area. Height of eelgrass beds from acoustic data was compared to that obtained by direct observation at 34 positions, and the measurement error was determined. First, we dropped underwater camera with rope in sea water to sea bottom from side of transducer. Then, we pulled the rope up until the underwater camera to the top of eelgrass beds by checking the video recorded. Heights of eelgrass beds ware defined as the pulling up length of rope. Measurement error of the height of eelgrass bed from acoustic data and direct observation was about 10 cm (Fig 4).

There is a possibility that threshold would change among varied points if big fish having big swim bladder exist in eelgrass beds when setting acoustic threshold of eelgrass beds. But, the animal inhabitants of eelgrass beds are very small, such as juvenile fish [10, 26], and acoustic reflections from these animals are very small and obscured in reflection of eelgrass beds.

### Spatial interpolation

We mapped presence/absence and height of eelgrass beds using ArcGIS10.1 (ESRI, USA). The height of the eelgrass beds was checked using the Tukey test in different seasons and areas. Additionally, we estimated the area of distribution of eelgrass beds between transect lines using Kriging [27], a spatial interpolation method for obtaining a value at a point without direct observation from neighboring observations using spatial autocovariance.

In this study, we used ordinary and probability Kriging [28]. Probability kriging was used to estimate the presence or absence of eelgrass beds, and ordinary kriging was used to estimate the height of eelgrass beds in the survey area. Then, the height of the eelgrass beds was extrapolated.

### Carbon sink and fixation

Typically, carbon sink and fixation are reported in units of production of gram dry weight per unit area per day (gdw m^-2^ d^-1^). Carbon sink and fixation were the same in areas of this study (Tables 2 and 3).

**Table 2:** Seasonal production and carbon sink in eelgrass beds per unit area/day^a^.

		Production of seagrass beds per area/day (gdw m^-2^ d^-1^)		Carbon sink of seagrass beds per area/day (g-C m^-2^ d^-1^)	
period		Average	±SD	Average	±SD
Declining period	(November)	1.75	0.29	0.57	0.09
Increase period	(May)	3.63	0.98	1.17	0.32
Yearly average		2.69	0.49	0.87	0.16

^a^ Data from Fisheries Research Agency (2011).

**Table 3:** Carbon fixation in eelgrass beds per unit area/day^a^.

	Dropleaves		Flow		Fixation of seagrass in area		Carbon fixation of seagrass beds per unit area/day	
	(gdw m^-2^ d^-1^)						(g-C m^-2^ d^-1^)	
Growth situation	Average	±SD	Average	±SD	Average	±SD	Average	±SD
Thick eelgrass beds (Cover degree 80~100%)	4.44	0.95	1.33	0.28	3.11	3.11	1.00	0.22
Thin eelgrass beds (Cover degree 40~50%)	2.28	0.71	1.60	0.50	0.68	0.68	0.22	0.07

^a^ Data from Fisheries Research Agency (2011).

This data shows carbon sink per unit area of eelgrass beds show a declining season (November), a growth season (June), and an average season (July and September), and carbon fixation per unit area of eelgrass beds show a thick area (North area) and a thin area (East area). Carbon sink and fixation per unit area in coastal waters near Ikunojima Island were reported for 2011 (gdw m^-2^ d^-1^) by the National Research Institute of Fisheries and Environment of Inland Sea [3] Seasonal summaries were determined and used to calculate carbon sink and fixation. To determine the carbon sink, they labeled the base of eelgrass plants. Then, they collected eelgrass plants labeled by pot cutting after about two weeks, and calculated growth amount of eelgrass per unit day. Carbon sink was calculated as the production per unit area and day each season (gdw m^-2^ d^-1^) based on carbon content of eelgrass of 32.3% [27].

Carbon sink=W ×R

“W” is growth amount of eelgrass per unit area of each season (gdw m-2 d^-1^), and “R” is carbon content ratio of the eelgrass leaves. Carbon sink per unit area of distribution was calculated from carbon sink per unit area.

Carbon fixation per unit area in coastal waters near Ikunojima Island were reported in 2011 (gdw m^-2^ d^-1^) by the National Research Institute of Fisheries and Environment of Inland Sea [3], too. To determine the carbon fixation, they estimated number of dropout plants based on the number of exist plants in growth period and declining period. Then, they calculated dropout amount per unit plant by subtracting the growth amount and dropout plant. Dropped outflow leaves are known to be 30% in thick eelgrass beds and 70% in the thin eelgrass beds [29]. The outflow amount to the outside of eelgrass beds became apparent through dropped amount and outflow amount. Carbon fixation was determined as the loss in weight due to leaves flowing to other areas from eelgrass beds (gdw m^-2^ d^-1^). First, we excluded the weight of leaves flowing to other areas (gdw m^-2^ d^-1^) from the production of eelgrass beds (gdw m^-2^ d^-1^). Then, carbon fixation was obtained based on a carbon content of 32.3%. Because carbon fixation is different in thick and thin eelgrass beds, we applied values for thick eelgrass beds in the North area where eelgrass grows prolifically and that for thin eelgrass beds in the East area where eelgrass is less prolific. Following equations is used to determine the amount of carbon to be fixed to the area.

Carbon fixation=R(D−F)

“D” is dropped eelgrass amount per unit area of each area (gdw m^-2^ d^-1^), “F” is amount of outflow leaves and “R” is carbon content ratio of the eelgrass leaves. We calculated carbon fixation per unit area from carbon fixation data.

Finally, carbon sink and fixation every survey area is calculated by multiplying carbon sink and fixation per unit area each season by estimated distribution area of eelgrass beds using kriging method.

## Results

### Distribution area

**Fig 5:**
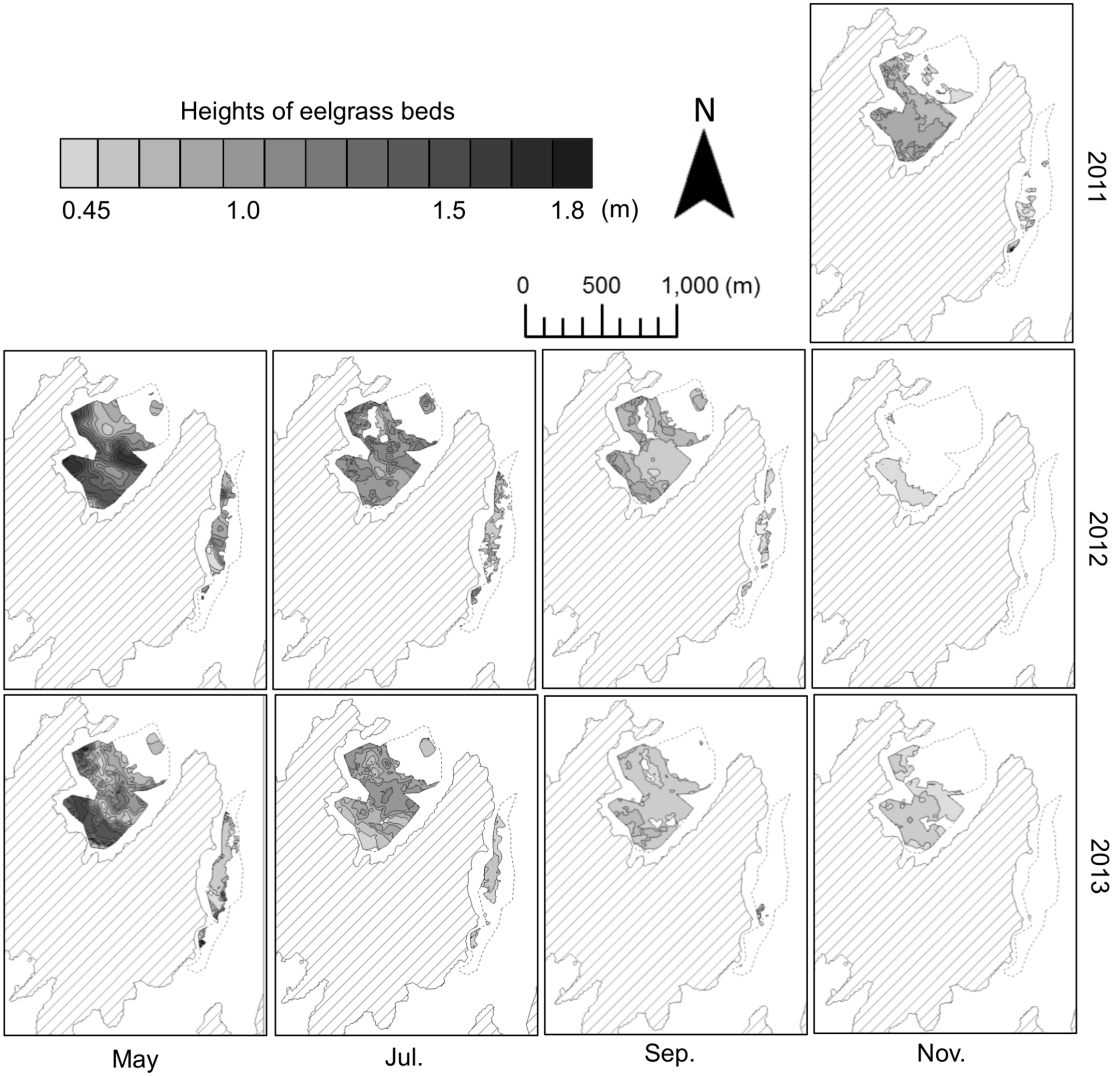
Estimated area of distribution of eelgrass beds. The data from survey years are shown top, 2011; middle, 2012; and bottom, 2013. Distributions are shown for the progression through seasons from left to right as follows: May, growth period; July, September and November, declining period. Height of eelgrass beds is indicated by color. Dashed line indicates the survey area set in this study.

Eelgrass beds were found only in water depths of less than 8 m, and all areas identified as having eelgrass beds were confirmed as such by direct observation. Fig 5 shows the area of distribution of eelgrass beds.

**Fig 6:**
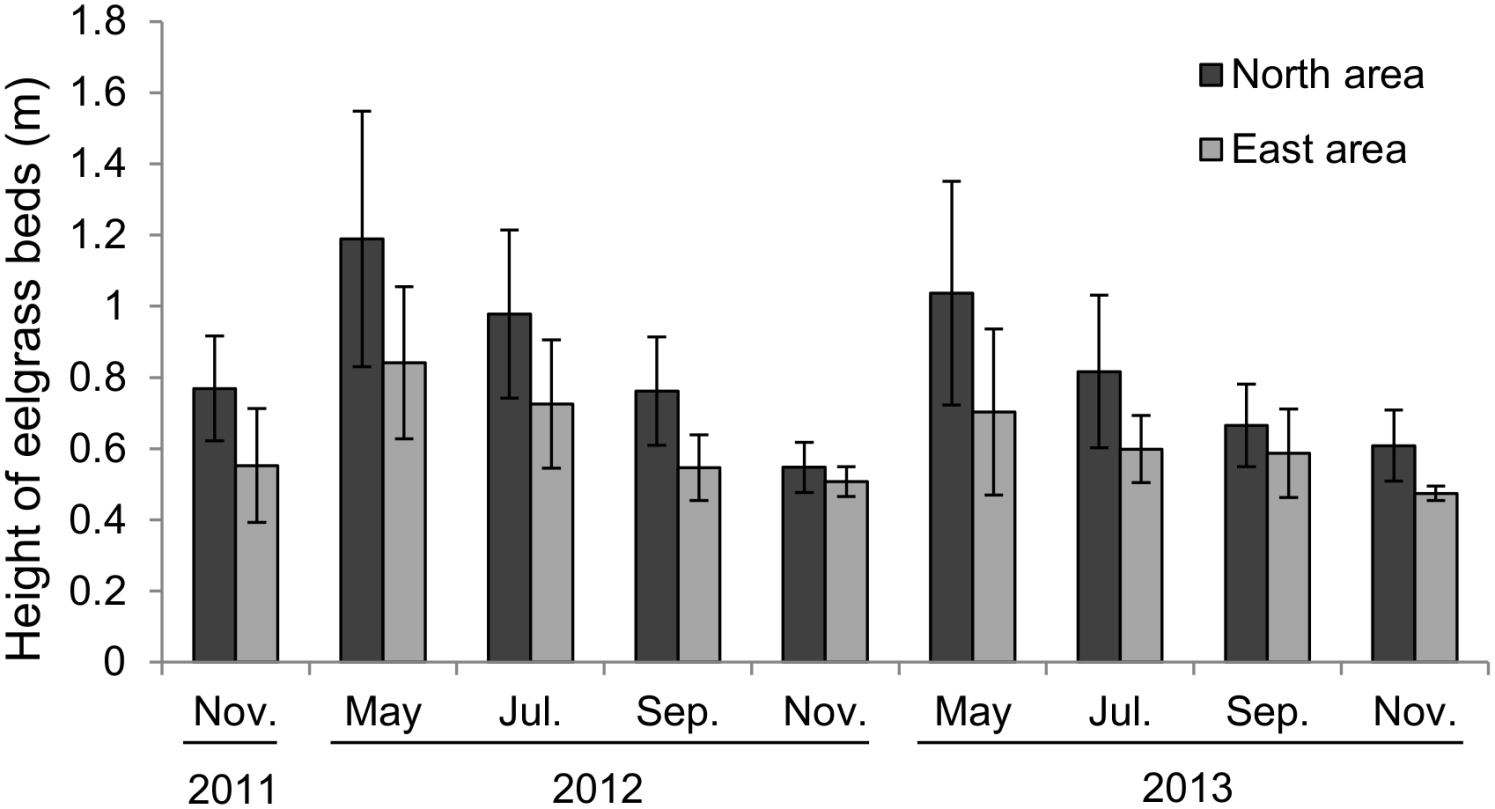
Mean height of eelgrass beds from acoustic data each season. Dark gray is north area, and pale gray is east area. Error bars indicate standard deviation.

The average height based on acoustic data showed seasonal patterns (Fig 6) with a peak in May of 1.01 ± 0.28 m in 2012 and 0.86 ±0.27 m in 2013 and a minimum in November with the following pattern: 0.66 ± 0.15 m in 2011, 0.52 ± 0.06 m in 2012 and 0.54 ± 0.06 m in 2013.

Eelgrass beds were significantly thicker in the North area than in the East area (Tukey, p<0.05). The eelgrass beds were relatively thicker in 2012 than in all other seasons in other years.

The area of distribution of eelgrass beds showed peak of the growth season in May (0.30 km^2^ in 2012 and 0.31 km^2^ in 2013) and a minimum value in the declining season in November (0.19 km^2^ in 2011, 0.05 km^2^ in 2012 and 0.16 km^2^ in 2013) (Table 4).

**Table 4:** Estimated area of distribution of eelgrass beds based on acoustic data.

		Distribution area (km^2^)		
Year	Month	North area	East area	All area
2011	Nov.	0.19	0.02	0.22
2012	May	0.25	0.05	0.30
	Jul.	0.21	0.04	0.25
	Sep.	0.23	0.03	0.26
	Nov.	0.05	0.00	0.05
2013	May	0.25	0.06	0.31
	Jul.	0.24	0.05	0.29
	Sep.	0.21	0.00	0.22
	Nov.	0.16	0.00	0.16

**Fig 7:**
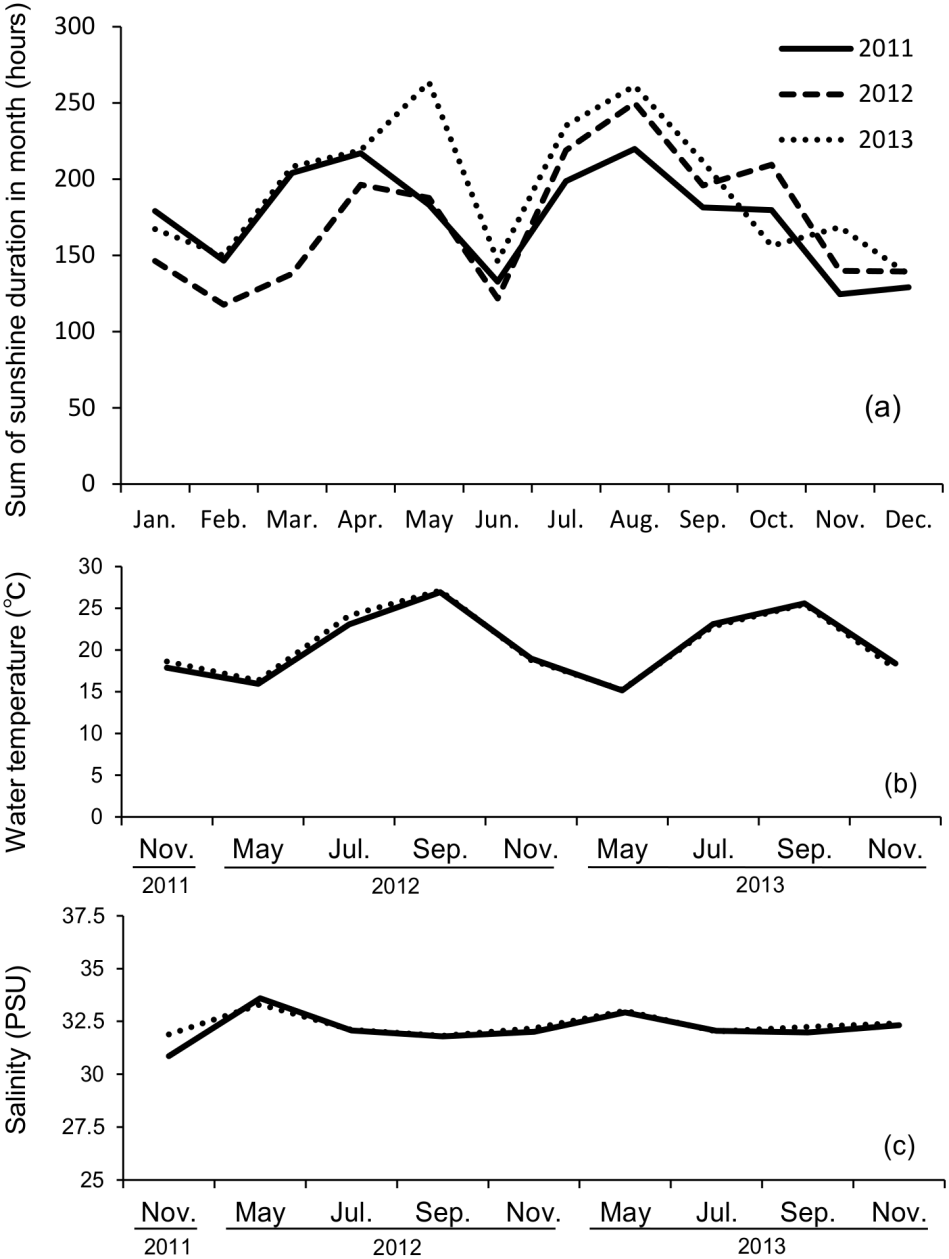
Environmental data for survey areas. (a) Monthly sunshine duration. (b) Mean monthly water temperature. (c) Mean monthly salinity. (b) and (C) shows North area, solid line; East area, dashed line. There were no significant differences for any of the parameters between survey areas (p>0.05).

In 2012, the area of distribution decreased drastically from September to November. Eelgrass beds had larger distribution in the North area than in the East area in all seasons. During the same period, sunshine duration, temperature and salinity showed no obvious differences between the North area and the East area (t-test, p>0.05, Fig 7).

### Carbon sink and fixation

We calculated the carbon sink and fixation over the area of distribution for each season and year (Tables 5 and 6).

**Table 5:** Carbon sink and area of distribution of eelgrass beds by survey period and area (S1 Fig).

		Carbonsink of seagrass beds per area/day^a^ (g-C m^-2^ d^-1^)		Distribution area (km^2^)			Carbon sink per day/distribution area (kg-C d^-1^)		
Year	Month			North area	East area	All area	North area	East area	All area
2011	Nov.	Declining period	0.57	0.19	0.02	0.22	109.80	11.82	121.63
2012	May	Increase period	1.17	0.25	0.05	0.30	288.23	62.96	351.19
	Jul.	Yearly average	0.87	0.21	0.04	0.25	193.49	37.37	230.86
	Sep.	Yearly average	0.87	0.23	0.03	0.26	128.56	16.69	145.25
	Nov.	Declining period	0.57	0.05	0.00	0.05	26.78	0.00	26.78
2013	May	Increase period	1.17	0.25	0.06	0.31	290.07	68.71	358.78
	Jul.	Yearly average	0.87	0.24	0.05	0.29	211.35	40.10	251.45
	Sep.	Yearly average	0.87	0.21	0.00	0.22	121.11	1.75	122.86
	Nov.	Declining period	0.57	0.16	0.00	0.16	89.84	0.00	89.84
Average							162.14	26.60	188.74

^a^ Carbon sink of eelgrass beds per unit area/day was used to calculate the carbon sink of the entire survey area based on area of distribution.

**Table 6:** Carbon fixation by survey period and area based on area of distribution (S2 Fig).

		Carbon fixation of seagrass beds per area/day^a^ (g-C m^-2^ d^-1^)		Distribution area (km^2^)			Carbon fixation per day/distribution area (kg-C d^-1^)		
Year	Month	Thick area	Thin area	North area	East area	All area	North area	East area	All area
2012	May	1.00	0.22	0.25	0.05	0.30	247.14	11.80	258.95
2013	May			0.25	0.06	0.31	248.72	12.88	261.61
Average							247.93	12.34	260.28

^a^ Carbon fixation for the thick area was applied to the North area and that for the thin area was applied to the East area.

Carbon sink and fixation in coastal waters near Ikunojima Island was highest in May and lowest in November each year. The highest carbon sink was in May 2013 (358.78±42.63 kg-C d^-1^) and the lowest was in November 2012 (26.78±4.42 kg-C d^-1^) in all surveys. Also, the carbon fixation in the North area, where the thick eelgrass beds were distributed, was larger in all seasons: carbon fixation was largest in North area (247.14±11.80 kg-C d^-1^in 2012 and 248.72±12.88 kg-C d^-1^ in 2013) and lowest in East area (11.80±3.72 kg-C d^-1^ in 2012 and 12.88±4.06 kg-C d^-1^ in 2013). The North area is an enclosed area and has greater carbon fixation than the East area. In addition, carbon sink and fixation was extremely low in November 2012, and area of distribution at that time was very small compared other years. This demonstrates that there is annual and seasonal variation in carbon sink and fixation in eelgrass beds.

## Discussion

The height and area of distribution of eelgrass beds varied year to year. The area of distribution declined drastically from September to November in 2012. Growth of eelgrass was limited by the photoenvironment, low precipitation, interaction among plants and disturbance of environmental factors [30]. There were no obvious differences in sunshine duration in the survey areas from 2011 to 2013 based on data of the Meteorological Agency (t-test, p>0.05). Tidal fluctuation was large in survey area, but eelgrass was unlikely to dry out, as it was not exposed to air at any time. There was almost no interaction among plants because the primary species was eelgrass and no other species were identified using an underwater camera in the survey area. Eelgrass has two modes of propagation: propagated with rhizomes and seed propagation. In the rhizome propagation method, branches are produced once or twice a year. In seed propagation, seeds that are produced are spread widely, expanding the range of the species. Newly produced eelgrass biomass would decline in the seed propagation process, as seeds become buried in the sand by wave action and sudden rain before becoming fixed on the sea bottom [31]. In coastal waters near Ikunojima Island, eelgrass blooms and produces seeds in May. A decreased in the biomass of eelgrass beds would occur if the disturbance occurs before these seeds become fixed. Southwesterly winds blew in 2011 and 2013, and northeasterly winds blew in 2012 as seed propagation ended and eelgrass started to decline in the summer. Waves would be large in the North area in the bay facing north and in the East area in the coast facing east when northeasterly winds are blowing. Eelgrass beds of biomass decreased in September 2012, and this led to a decrease in the area of distribution of the same year in November. The eelgrass beds showed a reduced area of distribution in November 2012 but the area of distribution recovered in the following growing season at the same scale in both areas but with lower bed height than in the previous year (Tukey, p>0.05). In the East area, the decrease in the area of distribution in September and November 2013 was drastic compared to that in the previous year. This indicates that the decrease in distribution due the effect of wind was strong on small and unstable eelgrass beds. For these reasons, wind disturbance has a strong effect on the new production of eelgrass biomass. Disturbance by wind or squall is considered as one of the important determinants for the height and spatial distribution of eelgrass beds and their annual variability [1].

Height of eelgrass beds differed between the North area and the East area. For example, the average height was 1.19 m in the North area, and 1.04 m in the East area in May 2012. The coastal marine environment was thought to be the main factor influencing the distribution of eelgrass beds between the North area and the East area. The water temperature and salinity were similar and sea bottom was covered with sand in areas where eelgrass grows in both areas, but the topography of the sea floor was different between the North area and the East area. The North area had a calm inner bay and the impact of waves was small. On the other hand, the East area was susceptible to wave impact compared to that of the North area as it was situated along an open coast line. The East area is in the open sea, and a ferry course operates at 300 to 500 m offshore. The North area is less influenced by waves and has better growing conditions than the East area. This result shows that the growth of eelgrass is affected by the shape of the coast along which it is situated.

The area of distribution differed in between the survey areas with the North area being larger than the East area. It is thought that this is due to in part to the great difference in water depth between the survey areas. Eelgrass has a distribution in coastal waters with a depth of 1 to 10 m [1]. In the North area, the area of distribution of eelgrass was 0.25 km^2^ and the water depth was less than 8 m in over 70% of this survey area. Eelgrass was distributed widely in the North area because of it being a wide shallow area. In the East area, eelgrass was distributed in coastal waters with a depth of less than 8 m. The distribution area was small (about 0.08 km^2^) as the sea bottom became drastically deeper. The sharp slope of the sea bottom was reported to block the growth of eelgrass [32]. Thus, the sharp slope was considered to be the reason for the lower distribution of eelgrass in the East area.

Carbon sink per unit area was largest in the growth season (May), and it showed the same trend in the area of distribution. The carbon sink of eelgrass beds was considered to be at a maximum value in May as both the value per unit area and the area of distribution were the largest. In particular, in the North area, although there are variations in the height and area of distribution of eelgrass beds due to seasonal fluctuations, the stable distribution as a community was also seen to decline in this period. Thus, the stability of the carbon supply source of eelgrass beds in the North area is higher than in the East area.

Carbon fixation per unit distribution area was higher in the north area. Many of the leaves were retained in the North area as it is a semi-closed bay. This indicated a higher value of carbon fixation due to the high eelgrass biomass. In the East area, many leaves were swept out of the area as it is an open coastal area, and it is the reason for low biomass and low carbon fixation. Much organic carbon taken up by eelgrass was fixed in the North survey area. With the advantage of having the preferred organic carbon and the preferred environment for microorganisms, it is easier to establish a trophic cascade based on microorganisms. This area is closed area and which has high productivity, thus the biodiversity is apparently more abundant due to the self-sufficiency of organic carbon cycle in this area.

We quantified the carbon sink and fixation methods based on the estimations of values from a past study. It is important to have data on the carbon dioxide absorption ability of eelgrass beds in order to discuss whether eelgrass beds are effective for carbon absorption in coastal and inner bay areas. We can assess the potential biodiversity of a sea area based on the supporting services by quantifying the carbon sink and fixation in eelgrass beds.

The height and distribution of eelgrass beds was determined using a small quantitative echo sounder in this study. Acoustic data of eelgrass beds over both study areas were obtained from onboard our research vessel in about 5 h, which is less time for data collection than for previous methods. In addition, we quantified ecosystem services over a wider region than was examined previously. Knowledge of carbon sinks and fixation ability in eelgrass beds serves as an important index for development of coastal areas when viewed from the perspective of the conservation of coastal ecosystems. It may be possible to discuss availability of eelgrass beds with respect to the economic impact on biomass production and fisheries.

Continuous monitoring of coastal environments, such as to assess the distribution of eelgrass beds is very important for understanding the current condition of biodiversity and for responding to needs for the preservation of coastal environments and fishery resources. Long-term data sets of conditions in eelgrass beds are necessary for considering seasonal variations in the future.

## Supporting Information

S1 Dataset Acoustic threshold in all survey. (XLSX)

S2 Dataset Information of direct observation point. (XLSX)

S1 Fig Seasonal variation of carbon sink per day/distribution area. (TIF)

S2 Fig Yearly variation of carbon fixation per day/distribution area. (TIF)
