# Supplementary figures and images for: Using Acoustics to Determine Eelgrass Bed Distribution and to Assess the Seasonal Variation of Ecosystem Service
Source: PLoS One. 2016 Mar 8;11(3):e0150890. doi: 10.1371/journal.pone.0150890 (PMC4783055; doi:10.1371/journal.pone.0150890)

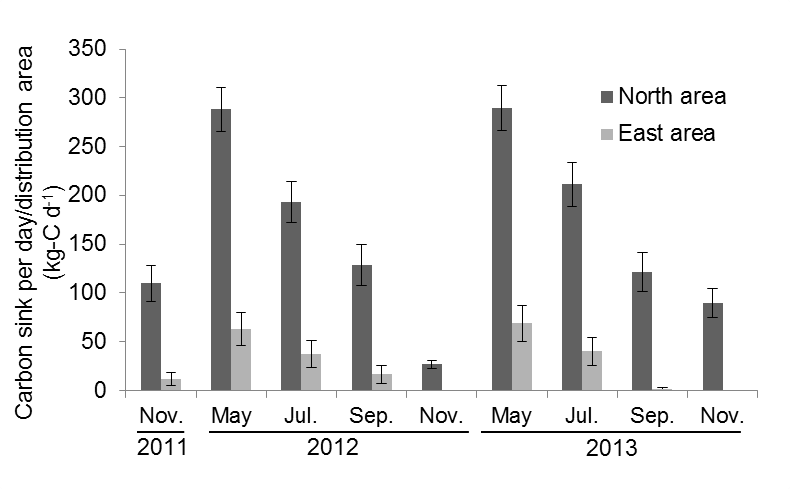

Supplement: S1 Fig — (TIF) [file pone.0150890.s003.tif]

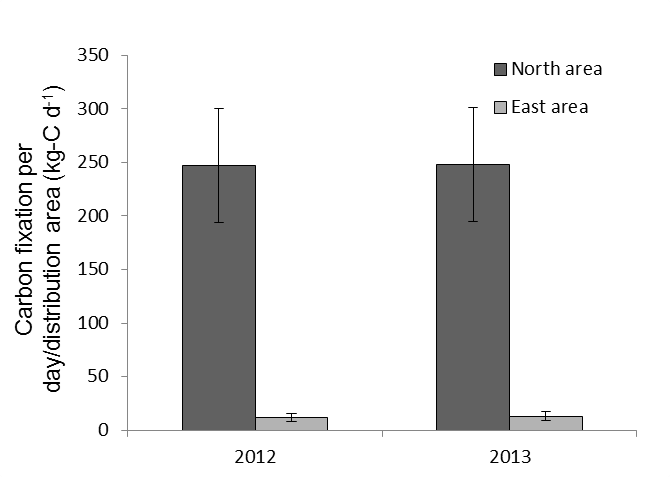

Supplement: S2 Fig — (TIF) [file pone.0150890.s004.tif]
